# Effect of electrical stimulation on patients with diabetes-related ulcers: a systematic review and meta-analysis

**DOI:** 10.1186/s12902-022-01029-z

**Published:** 2022-04-27

**Authors:** Yinhua Zheng, Xue Du, Liquan Yin, Hongying Liu

**Affiliations:** 1 Department of Rehabilitation Medicine, The First Hospital of Jilin University, No.1, Xinmin Street, Chang Chun, 130021 Jilin Province China; 2 Department of Clinical Laboratory Medicine, Affiliated Hospital, Changchun University of Chinese Medicine, Changchun, 130021 China; 3 Department of Rehabilitation Medicine, The Third Hospital of Jilin University, Changchun, 130033 China

**Keywords:** Diabetes-related ulcers, Electrical stimulation, meta-analysis, Systematic review, Ulcer healing

## Abstract

**Background:**

This study aimed to systematically review the literature to better understand the efficacy of electrical stimulation (ES) for the treatment of patients with diabetes-related ulcers.

**Methods:**

We searched the Embase, Medline, and Cochrane Library databases through July 31, 2021. Original trials for ES treatment of patients with diabetes-related ulcers with placebo or standard care as the control group were included. The primary outcomes were ulcer area reduction and healing rates. Meta-analyses were performed to compare the standardized mean difference (SMD) in the percentage of ulcer reduction and risk ratio of non-healing rates between ES treatment and placebo or standard care. We used the Revised Cochrane risk-of-bias tool for randomized trials to assess the risk of bias for each included article. Funnel plots and Egger’s test were used to assess publication bias.

**Results:**

Compared to placebo or standard care, ES had a significant benefit for the treatment of patients with diabetes-related ulcers in terms of percentage of ulcer reduction (SMD = 2.56, 95% CI: 1.43–3.69; *P* < 0.001 (Q-test), *I*^2^ = 93.9%) and ulcer healing rates [risk ratio of non-healing rates for the ES group was 0.72 (95% CI: 0.54–0.96; *P* = 0.38 (Q-test), *I*^2^ = 2.3%)]. Two, four, and three of the included studies were categorized into low risk of bias, some concerns, and high risk of bias, respectively. No publication bias was found.

**Conclusions:**

Based on the findings of this meta-analysis, ES could be used to treat patients with diabetes-related ulcers. ES treatment was effective for ulcer area reduction and ulcer healing, although it had a high heterogeneity level among the included studies. Pulsed current ES has the potential benefit of increasing ulcer healing compared to direct current ES. Further large-scale clinical trials are needed to define the adverse events and potentiators of ES in the treatment of patients with diabetes-related ulcers.

**Supplementary Information:**

The online version contains supplementary material available at 10.1186/s12902-022-01029-z.

## Background

In 2019, it was reported that 463 million people had diabetes worldwide, and this number is expected to reach 693 million by 2045 [1]. Diabetes-related neuropathy, peripheral arterial disease, and infection can lead to foot and lower leg ulcers, which can significantly impair a patient’s quality of life [2, 3]. Approximately 6.3% of patients with diabetes have foot ulcerations, and the prevalence of leg ulceration is approximately 1 to 2% [4, 5]. These ulcers often recur after healing and are associated with a high risk of amputation and death, as well as high medical expenses [2].

Debridement, negative pressure wound therapy, and antibacterial treatment are essential in the management of diabetes-related foot ulceration [2, 6]. However, these treatments are not always effective for all patients. Therefore, practitioners and researchers have been looking for alternative adjuvant treatments.

Electrical stimulation (ES) is a physical therapy modality that sends gentle electrical pulses through the skin [7]. The technique has been widely used in pain management and wound healing [8–10]. ES treatment is advantageous because it is cost-effective, simple, and has few complications. A recent meta-analysis showed that ES may be an effective adjunctive therapy for accelerating diabetes-related foot ulceration healing [11]. However, there is a lack of systematic evidence of the efficacy of ES for all diabetes-related ulcers. The objective of the present study was to systematically review the literature to better understand the efficacy of ES for the treatment of patients with diabetes-related ulcers.

## Materials and methods

The reporting of the present review followed the PRISMA statement for systematic reviews and meta-analyses [12, 13]. The ethics review was waived because of the retrospective and anonymous characteristics of the study.

### Literature search and study selection

We searched the Embase, Medline, and Cochrane Library databases through July 31, 2021. Search terms included “electric stimulation”, “electric stimulation therapy”, “transcutaneous electric nerve stimulation”, “skin ulcer”, and “ diabetes related ulcers”. The search strategies are provided in the Additional file 1: Appendix 1. The inclusion criteria of the articles were as follows: 1) randomized controlled trials (RCTs) and quasi-experimental studies in English, 2) patients with diabetes-related ulcers, including foot and leg ulcers, 3) ES as intervention, 4) having placebo or standard care as a control group, and 5) having information of target outcomes of healing rates or ulcer area reduction rates. The exclusion criteria were as follows: 1) not an original study (e.g., reviews, protocols, letters, or commentaries), 2) animal (non-human) studies, and 3) studies without information on target outcomes.

We included all ES types in the present review, including pulsed current type and direct current type, as the experiment arm. The primary outcomes were ulcer area reduction and healing rates. Other terms of standard care for the eligible control treatment were “usual care” and “standard treatment.”

After omitting duplicated studies, two independent reviewers (DX and ZYH) screened the titles and abstracts according to the eligibility criteria and defined the list of articles for full-text review. Title and abstract screening were performed using Endnote X_9_. The pre-test review form was used for the full-text review by the same independent reviewers. The reasons for excluding papers were discussed in detail between the reviewers. Any inconsistency regarding article inclusion was solved by discussion or with the help of a third-party reviewer (ZYH).

### Data collection

YLH and LHY performed the data collection independently using the pre-test data collection sheet. The primary characteristics of the included studies - demographic characteristics of participants, sample size, countries, publication year, ES types, and follow-up period - were collected. Data of the two arms were collected for sample size, sex, and percentage of ulcer area reduction or ulcer healing.

### Data analysis

All data analyses were performed using Stata, version 15.0 (Stata Corp. Texas, USA). Categorical variables are expressed as count and percentage/proportion. Continuous variables are expressed as mean with standard deviation (SD). Meta-analysis was performed for outcome measures. One of the primary outcomes was healing rate, which was calculated as the number of healing patients divided by total patients. Healing rates are presented as the risk ratio (RR) of non-healing rates with a 95% confidential interval (CI), and ulcer area reduction is presented as the standardized mean difference (SMD) with 95% CI. Heterogeneity was estimated using the Q-test and *I*^2^ score. When the *P*-value was < 0.1 (for Q-test) and *I*^2^ > 50%, the result was considered with heterogeneity, and the random-effects model was used for analysis. Otherwise, a fixed-effects model was applied for analysis. A *P*-value of < 0.05 was set as the threshold for statistical significance. Subgroup analysis was performed according to the ES types.

As limited data were available, meta-regression could not be performed. We evaluated the risk of bias using the Revised Cochrane risk-of-bias tool for randomized trials (RoB2) [14]. Funnel plots and the Egger’s test were used to determine publication biases.

## Results

**Fig. 1:**
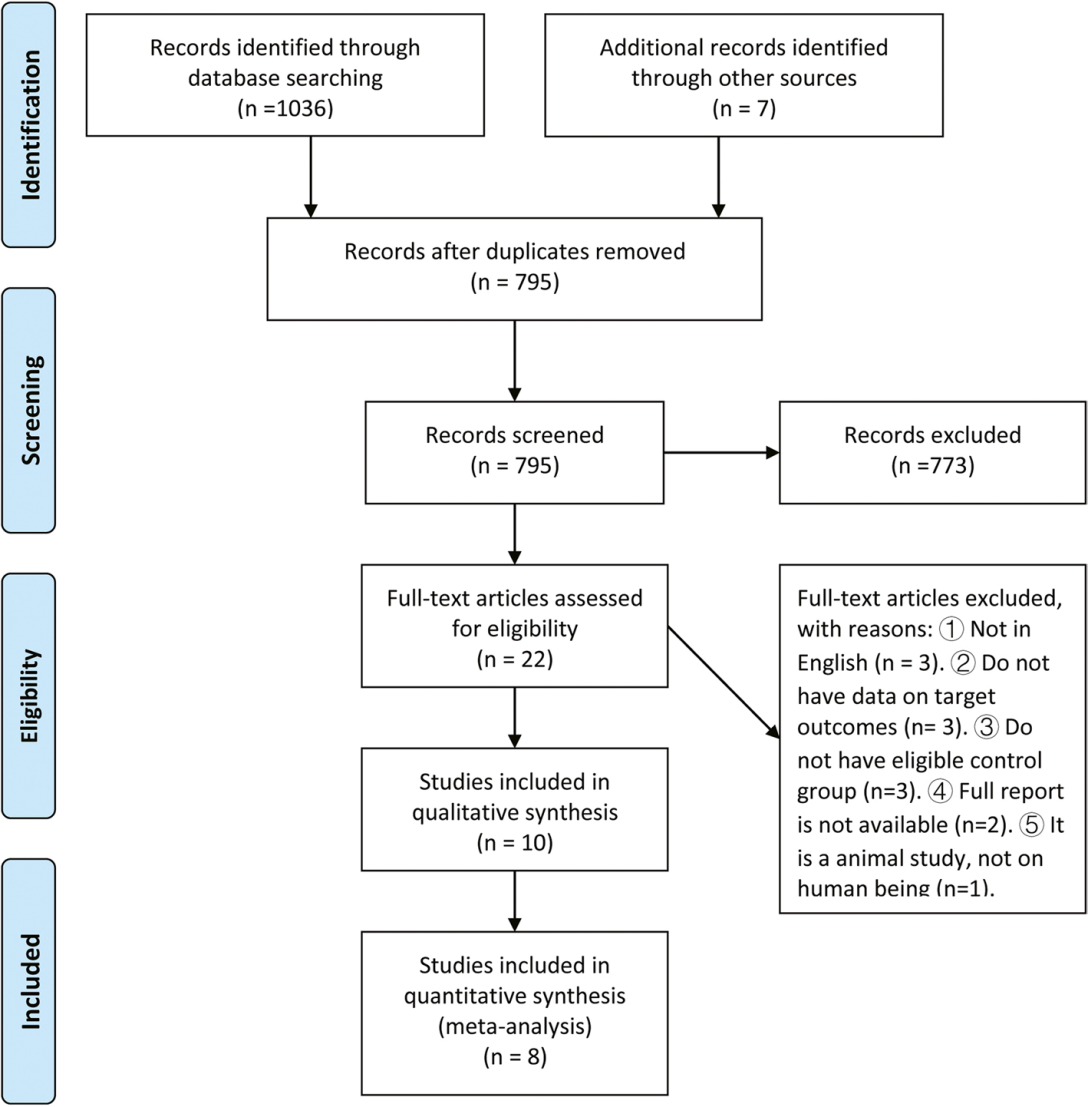
PRISMA flow diagram for article selection for meta-analysis

A total of 1042 articles were identified using our search criteria. After omitting duplicated studies, 794 articles were further screened for title and abstract. Of these, 20 articles were selected for full-text review. Finally, 10 articles were included in the data quality assessment and data analysis [15–24]. Study inclusion is present in Fig. 1.

### Characteristics of the included studies

All studies were RCTs published from 1992 to 2021. Our analysis included a total of 352 patients with diabetes-related ulcers ranging in age from 48.4 to 65.1 years. The median follow-up period ranged from 4 to 12 weeks. There were two experimental groups with different adjuvant heat plus ES [18], three studies reported direct current ES as the treatment modality [22–24], and all other studies applied pulsed current ES as the intervention. Details of the study characteristics are described in Table 1.

**Table 1:** Characteristics of included articles

Author, publication year, and Country	RCT Design	Participants	Sample size		Age (years), mean ± SD		Male, n (%)	ES type	Length of follow up (weeks)
			ES arm, n	Control arm, n	ES arm	Control arm			
Lundeberg et al. 1992, Sweden [15]	Parallel, placebo	Patients with diabetic leg ulcers	24	27	67.5 ± 8.6	66.0 ± 7.9	36 (70.6)	Pulsed current	12
Baker et al. 1997, USA [16]	Parallel, no placebo	Patients with diabetic open ulcers	21	20	58.0 ± 2.0	52.0 ± 2.0	41 (67.2)	Pulsed current (symmetric biphasic)	8
			20		50.0 ± 2.0			Pulsed current (square-wave pulse)	
Peters et al. 2001, Netherlands [17]	Parallel, placebo	Patients with diabetic foot ulcers	18	17	54.4 ± 12.4	59.9 ± 7.0	32 (91.4)	Pulsed current	12
Petrofsky et al. 2007, USA [18]	Parallel, placebo	Patients with diabetic foot or leg ulcers	10	10	64.7 ± 13.2	63.0 ± 7.6	Not specified	Pulsed current+ global heat	12
			9		62.0 ± 7.7			Pulsed current+ local heat	
Petrofsky et al. 2010, USA [19]	Parallel, no placebo	Patients with foot chronic diabetic ulcers	10	10	48.4 ± 14.6		Not specified	Pulsed current	4
Liani et al. 2014, Italy [20]	Parallel, no placebo	Patients with diabetic foot ischemic lesions	29	27	Not specified	Not specified	Not specified	Pulsed current	7
Mohajeri-Tehrani et al. 2014, Iran [23]	Parallel, placebo	Patients with diabetic foot ulceration	10	10	57 ± 3.2	56.1 ± 2.9	17 (85.0)	direct current	4
Ortíz et al. 2014, Colombia [21]	Parallel, no placebo	Patients with diabetic distal legs or feet ulcers	10	9	Not specified	Not specified	Not specified	Pulsed current	9
Asadi et al. 2017, Iran [22]	Parallel, no placebo	Patients with ischemic diabetic foot ulcerations	13	11	60.8 ± 5.5	60.1 ± 6.4	14 (58.3)	Direct current	4
Zulbaran-Rojas et al. 2021, USA [24]	Parallel, placebo	Patients diagnosed with diabetes mellitus type 2 with chronic non-healing wounds	16	17	65.1 ± 13.8	61.4 ± 11.2	21 (61.8)	Direct current	Not specified

*Abbreviation*: *SD* Standard deviation, *ES* Electrical stimulation, *RCT* Randomized controlled trial

### Risk of bias assessment

None of the included studies had the bias of missing data. Seven of the nine included RCTs (7/10 studies, 70.0%) had some concerns of bias in selecting reported results due to lack of protocol information or trial registration. Five studies had some concerns about risk in randomization because there was no information in random sequence generation (6/10 studies, 60.0%). Three studies had some concerns in both deviations from intended intervention and measurement of the outcome due to the risk in allocation concealment or blinding of participants and personnel (3/10 studies, 30.0%). In summary, two studies [21, 22], four studies [15–17, 23], and four studies [18–20, 24] were categorized into low risk of bias, some concerns, and high risk of bias, respectively. The details of the risk of bias assessment can be found in Table 2.

**Table 2:**
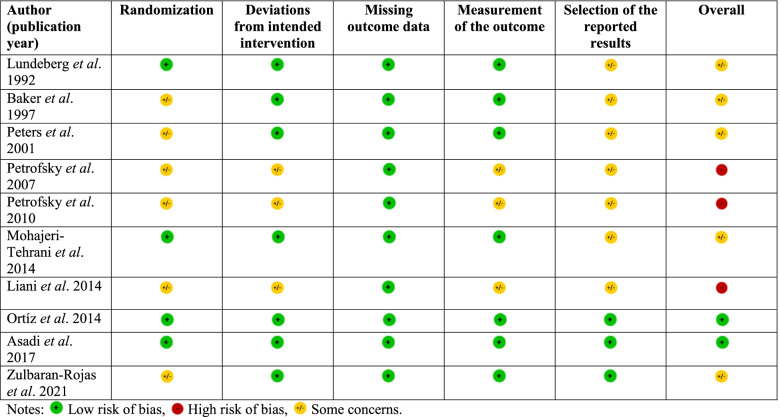
Summary assessment of risk of bias for included studies using the revised Cochrane risk-of-bias tool for randomized trials (RoB2)

### Ulcer area reduction

**Fig. 2:**
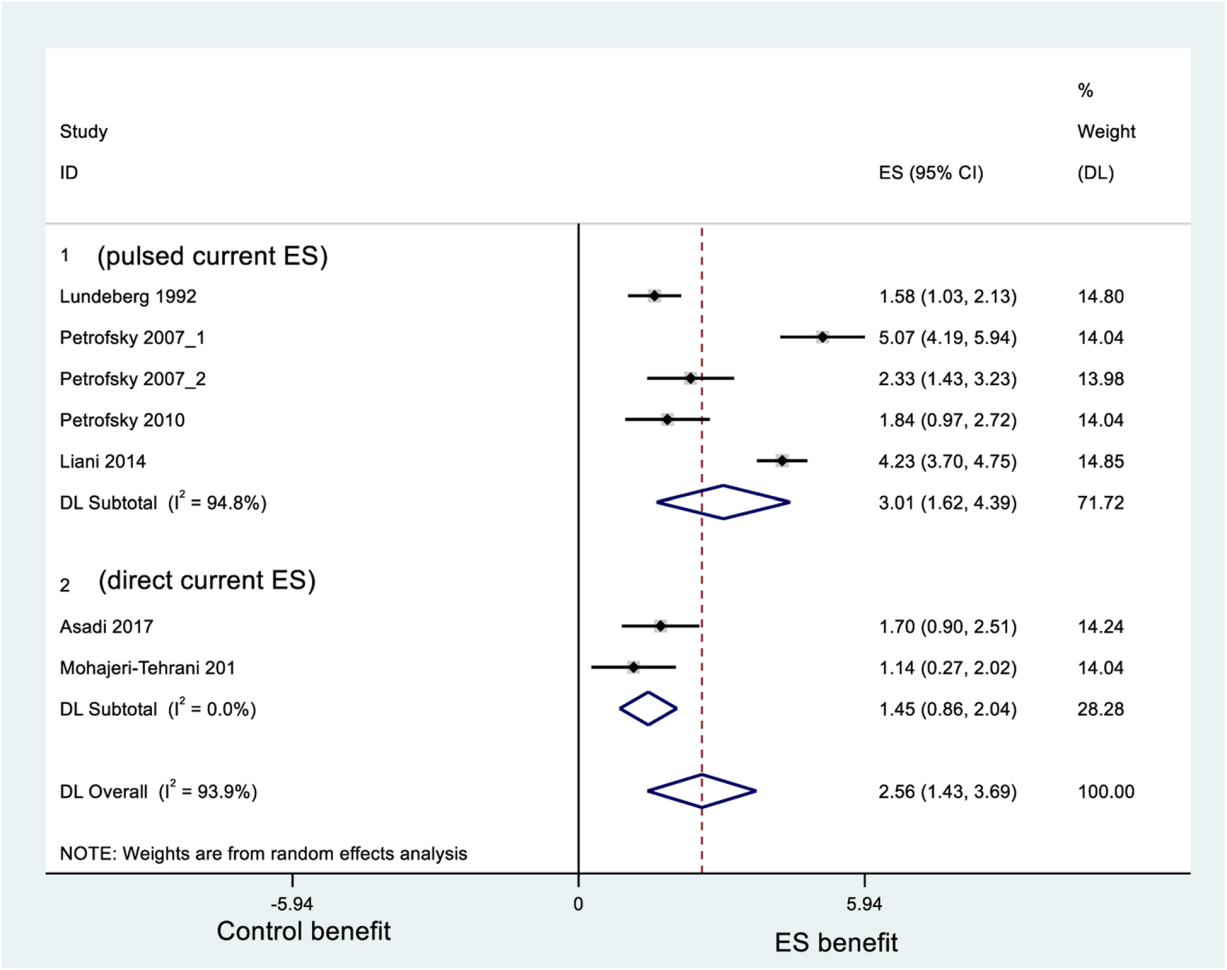
Forest plot of percentage reduction of diabetes-related ulcer area for electrical stimulation (ES) in standardized mean difference (SMD)

The ES arm of our meta-analysis showed statistical benefit for ulcer area reduction compared to the control arm (Fig. 2). The percentage of ulcer area reduction was significantly greater in patients treated with ES than in those treated with standard care or placebo (SMD = 2.56, 95% CI: 1.43–3.69; *P* < 0.001 (Q-test), *I*^2^ = 93.9%). See data extraction results in Additional file 2: Appendix 2.

According to the subgroup analysis of current types, there was no statistical difference in ulcer area reduction between pulsed current ES (SMD = 3.01, 95% CI: 1.43–3.69) and direct current ES (SMD = 1.45, 95% CI: 0.86–2.04). The highest ulcer reduction was found in Petrofsky et al. (2007) with global heat plus ES. According to the subgroup analysis of ulcer types, there was no statistical difference in ulcer area reduction between leg ulcers (SMD = 1.58, 95% CI: 1.03–2.13) and foot ulcers (SMD = 2.41, 95% CI: 0.88–3.94). After omitting the studies with serious risk or some concerns of bias, the percentage of ulcer area reduction was still significantly greater in patients treated with ES than in those treated with standard care or placebo (SMD = 1.52, 95% CI: 1.11–1.92). The study reported by Zulbaran-Rojas et al. does not have available data to enter the meta-analysis. However, the four-week ulcer deduction was significant in the ES arm (*P* = 0.002) but not in the control placebo arm (*P* = 0.982) [24].

### Healing rates

**Fig. 3:**
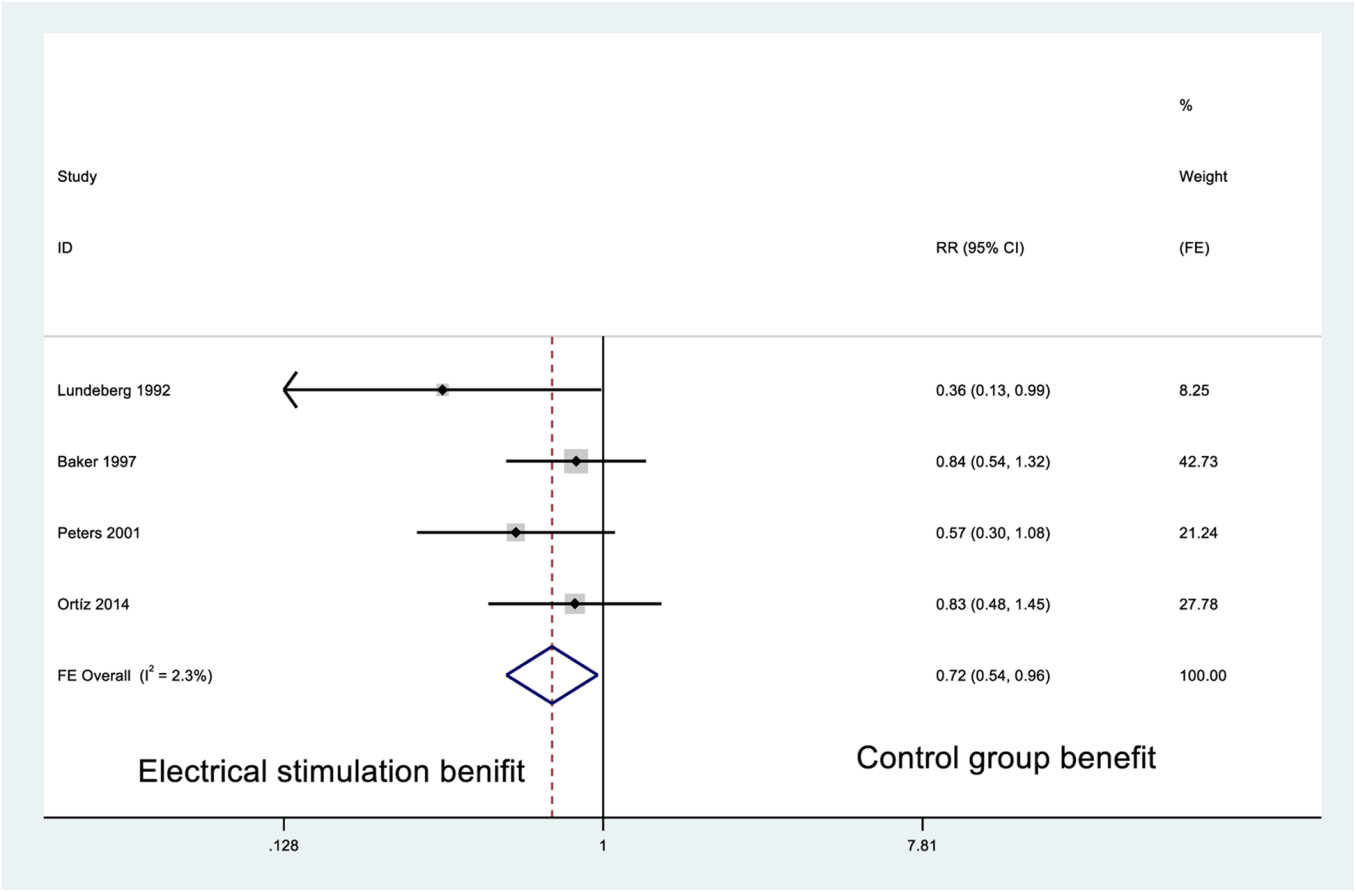
Forest plot of non-healing rates of diabetes-related ulcer in risk ratio

According to the pooled results, the diabetes-related ulcer healing rate was significantly higher in the ES arms than in the control arm (*P* < 0.05). Compared to the control group, the RR of non-healing rates for the ES group was 0.72 (95% CI: 0.54–0.96; *P* = 0.38 (Q-test), *I*^2^ = 2.3%; Fig. 3). According to the subgroup analysis of ulcer types, there was no statistical difference in healing rates between leg ulcers (0.36, 95% CI: 0.13–0.99) and foot ulcers (SMD = 0.71, 95% CI: 0.47–1.07). Only one study had risk of bias in the related data and was not about to perform sensitivity analysis. See data extraction results in Appendix 2.

### Publication bias

**Fig. 4:**
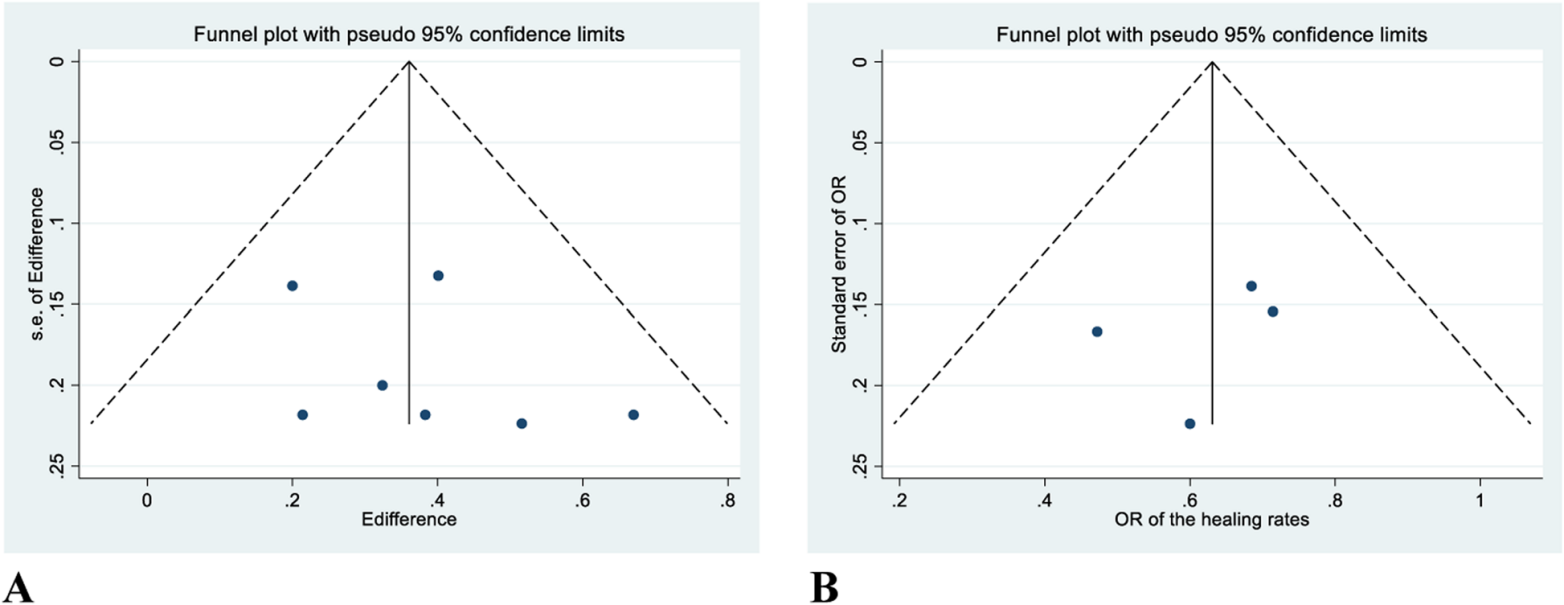
Funnel plots of A) ulcer reduction area and B) ulcer healing rates

The *P*-values of the Egger’s test for the percentage of ulcer area reduction and ulcer healing rates were 0.636 and 0.843, respectively. There was no obvious asymmetry in the funnel plots (Fig. 4), and Egger’s test suggested no publication bias although the number of studies was limited.

## Discussion

In the present systematic review and meta-analysis, we found that ES has a significant benefit compared to placebo or standard care for the treatment of patients with diabetes-related ulcers with regards to the percentage of ulcer reduction (SMD = 2.79, 95% CI: 1.58–4.01) and ulcer healing rates (RR of non-healing rates for the ES group was 0.72, 95% CI: 0.54–0.96). We applied the random effect model to the meta-analysis of SMD in the percentage of ulcer reduction due to the study heterogeneity (*P* < 0.001 (Q-test), I^2^ = 93.9%). The sources of heterogeneity may be due to the different ulcer types and follow-up times. Our findings are similar to a recently published systematic review on ES for people with diabetes-related foot ulcers [11]. Again, the slight difference in effect size between our results and the review may be due to different follow-up periods and the defined outcomes. We also included all ulcer types, including leg ulcers, in this meta-analysis.

It has been reported in preclinical studies that ES can enhance cellular activities (e.g., DNA and collagen synthesis, generation of chemotaxis factor, and adenosine triphosphate concentration) [25, 26]. In addition, ES can promote wound healing by increasing tissue perfusion and enhancing angiogenesis [27, 28]. Based on the findings of the present study, ES can be used as a critical treatment for people with diabetes-related ulcers in clinical practice.

ES can be categorized into direct current types: alternating current type and pulsed current type. Pulsed current ES involves a 1 ms to 1 s electrical flow as a monophasic pulsed waveform. In contrast, direct current ES uses unidirectional flow for 1 s or longer. Low frequency alternating current ES has not been used in wound healing, but the other two techniques have been explored [29]. In the present study, we did not find a statistical difference between the two ES types for ulcer healing and ulcer area reduction. However, the mean SMD of pulsed current ES (3.01) was greater than that of direct current ES (1.45), indicating that pulsed current ES may promote better healing of ulcers than direct current ES. Future large-scale clinical trials are needed to confirm this hypothesis.

Studies have shown a promising result of ES on wound recovery in diabetes-related ulcers on diabetic fibroblasts in diabetic animals [30, 31]. The rationale may be that ES functions to accelerate angiogenesis and enhance epithelialization [23, 32]. ES can also enhance sensation in diabetic neuropathic patients using mechanical noise [33–35] and significantly improve ischemic diabetic foot ulcers [22, 36]. However, we do not have enough data in the present study to explore the effects of ES on different diabetes-related ulcers. Future research is needed to provide more valid evidence (e.g., using ankle-brachial pressure index (ABPI) or vibration perception threshold (VPT) testing) during ES treatment to confirm its reliability [37, 38].

Some limitations of the present study should be noted. 1) ES appeared safe for treating people with diabetes-related ulcers. Therefore, we did not have sufficient data to explore the adverse events of ES. 2) We could not perform a meta-regression analysis to explore possible potentiators of ES effects due to the limited data. 3) There was insufficient data to perform subgroup analysis according to different ulcer types, which will be addressed in our future research. 4) Although all included studies were RCTs, only two studies were categorized as having a low risk of bias. The main limitations focus on lack of protocol information to clarify, no selection reporting, no information on the generation of randomization, and some concerns in blinding or masking. Therefore, the low quality of the evidence could potentially reduce the impact of the present findings.

## Conclusion

Based on the findings of this meta-analysis, ES could be used to treat people with diabetes-related foot or leg ulcers. ES treatment was effective for ulcer area reduction and ulcer healing, although it had a high heterogeneity level among the included studies. Pulsed current ES has the potential benefit of increasing ulcer healing compared to direct current ES. Further large-scale clinical trials are needed to define the adverse events and potentiators of ES in the treatment of people with diabetes-related foot or leg ulcers.

## Supplementary Information

**Additional file 1: Appendix 1.** Search strategy.

**Additional file 2: Appendix 2.** Data collection table.

## Data Availability

The datasets generated and/or analyzed during the current study are not publicly available since none of the data types requiring uploading to a public repository are contained in this manuscript but are available from the corresponding author on reasonable request.

## Abbreviations

ES: Electrical stimulation
SD: Standard deviation
CI: Confidential interval
SMD: Standardized mean difference
