# Supplementary material for: Effect of electrical stimulation on patients with diabetes-related ulcers: a systematic review and meta-analysis
Source: BMC Endocr Disord. 2022 Apr 27;22:112. doi: 10.1186/s12902-022-01029-z (PMC9044601; doi:10.1186/s12902-022-01029-z)
Supplement: Supplementary file 1 — Additional file 1: Appendix 1. Search strategy. [file 12902_2022_1029_MOESM1_ESM.docx]

**Appendix 1. Search strategy.**

**The Cochrane Central Register of Controlled Clinical Trials (CENTRAL)**

**Date Run: 29/03/2021 04:12:11**

**ID Search Hits**

**#1 MeSH descriptor: [Electric Stimulation] explode all trees 1907**

**#2 MeSH descriptor: [Electric Stimulation Therapy] explode all trees 7134**

**#3 MeSH descriptor: [Transcutaneous Electric Nerve Stimulation] explode all trees 1948**

**#4 (electric* next stimula*):ti,ab,kw 8552**

**#5 (electric* near/3 current*):ti,ab,kw 1037**

**#6 ("transcutaneous electric nerve stimulation" or "transcutaneous electrical nerve stimulation" or "transcutaneous electric stimulation" or "transcutaneous electrical stimulation"):ti,ab,kw 2837**

**#7 ((direct or puls*) next current*):ti,ab,kw 4461**

**#8 (("TENS" or "ES" or "ENS") near/5 (electric*)):ti,ab,kw 1614**

**#9 ((monophasic* or biphasic*) next (pulse or current*)):ti,ab,kw 63**

**#10 (("high frequency" or "low frequency") next (current*)):ti,ab,kw 43**

**#11 {or #1-#10} 18367**

**#12 MeSH descriptor: [Skin Ulcer] explode all trees 2920**

**#13 ((diabet*) next (ulcer*)):ti,ab,kw 247**

**#14 #13 or #12 3051**

**#15 #11 and #14 62**

**Database: OVID Medline Epub Ahead of Print, In-Process & Other Non-Indexed Citations, Ovid MEDLINE(R) Daily and Ovid MEDLINE(R) 1946 to Present**

**Search Strategy:**

**--------------------------------------------------------------------------------**

**1 exp Electric Stimulation/ (127383)**

**2 exp Electric Stimulation Therapy/ (82655)**

**3 exp Transcutaneous Electric Nerve Stimulation/ (8966)**

**4 electric* stimula*.tw. (53745)**

**5 (electric* adj3 current*).tw. (9908)**

**6 (transcutaneous electric* nerve stimulation or transcutaneous electric* stimulation).tw. (2877)**

**7 ((direct or puls*) adj current*).tw. (11475)**

**8 (("ES" or "ENS" or "TENS") adj5 electric*).tw. (3061)**

**9 ((monophasic* or biphasic*) adj (pulse or current*)).tw. (500)**

**10 ((high frequency or low frequency) adj current*).tw. (503)**

**11 or/1-10 (240499)**

**12 exp Skin Ulcer/ (46105)**

**13 (diabet* adj (ulcer* or sore* or injur*)).tw. (969)**

**14 12 or 13 (46617)**

**15 11 and 14 (328)**

*******************************

**Database: Embase <1974 to 2021 March 29>**

**Search Strategy:**

**--------------------------------------------------------------------------------**

**1 exp Electric Stimulation/ (70920)**

**2 exp Electric Stimulation Therapy/ (247320)**

**3 exp Transcutaneous Electric Nerve Stimulation/ (2265)**

**4 electric* stimula*.tw. (63914)**

**5 (electric* adj3 current*).tw. (10710)**

**6 (transcutaneous electric* nerve stimulation or transcutaneous electric* stimulation).tw. (3836)**

**7 ((direct or puls*) adj current*).tw. (13970)**

**8 (("ES" or "ENS" or "TENS") adj5 electric*).tw. (4126)**

**9 ((monophasic* or biphasic*) adj (pulse or current*)).tw. (634)**

**10 ((high frequency or low frequency) adj current*).tw. (484)**

**11 or/1-10 (340308)**

**12 exp Skin Ulcer/ (74646)**

**13 (diabet* adj (ulcer* or sore* or injur*)).tw. (1462)**

**14 12 or 13 (75256)**

**15 11 and 14 (646)**

*******************************
