# Supplementary material for: Effect of electrical stimulation on patients with diabetes-related ulcers: a systematic review and meta-analysis
Source: BMC Endocr Disord. 2022 Apr 27;22:112. doi: 10.1186/s12902-022-01029-z (PMC9044601; doi:10.1186/s12902-022-01029-z)
Supplement: Supplementary file 2 — Additional file 2: Appendix 2. Data collection table. [file 12902_2022_1029_MOESM2_ESM.doc]

**Appendix 2.** Data collection table.

| **Author, publication year, and Country** | Experimental group | | | | Control group | | | |
| --- | --- | --- | --- | --- | --- | --- | --- | --- |
|  | Sample size, n | Ulcer area reduction rate | Standard deviation of Ulcer area reduction rate | The number of non-healings | Sample size, n | Ulcer area reduction rate | Standard deviation of Ulcer area reduction rate | The number of non-healings |
| Lundeberg *et al*. 1992, Country not reported | 24 | 0.59 | 0.11 | 10 | 27 | 0.39 | 0.14 | 4 |
| Baker *et al*. 1997, USA | 21 |  |  | 15 | 20 |  |  | 12 |
| Peters *et al*. 2001, Netherlands | 18 |  |  | 13 | 17 |  |  | 7 |
| Petrofsky *et al*. 2007, USA | 10 | 0.707 | 0.169 |  | 10 | 0.037 | 0.08 |  |
| Petrofsky *et al*. 2010, USA | 9 | 0.553 | 0.312 |  | 10 | 0.037 | 0.08 |  |
| Liani *et al*. 2014, Italy | 10 | 0.684 | 0.286 |  | 10 | 0.301 | 0.067 |  |
| Mohajeri-Tehrani *et al*. 2014, Iran | 29 | 0.452 | 0.125 |  | 27 | 0.051 | 0.043 |  |
| Ortíz *et al*. 2014, Colombia | 10 |  |  | 8 | 9 |  |  | 6 |
| Asadi *et al*. 2017, Iran | 13 | 0.595 | 0.214 |  | 11 | 0.271 | 0.157 |  |
| Zulbaran-Rojas *et al*. 2021 | 10 | 0.31 | 0.2513 |  | 10 | 0.096 | 0.083 |  |
